# Effectiveness of In-Hospital Cholecalciferol Use on Clinical Outcomes in Comorbid COVID-19 Patients: A Hypothesis-Generating Study

**DOI:** 10.3390/nu13010219

**Published:** 2021-01-14

**Authors:** Sandro Giannini, Giovanni Passeri, Giovanni Tripepi, Stefania Sella, Maria Fusaro, Gaetano Arcidiacono, Marco Onofrio Torres, Alberto Michielin, Tancredi Prandini, Valeria Baffa, Andrea Aghi, Colin Gerard Egan, Martina Brigo, Martina Zaninotto, Mario Plebani, Roberto Vettor, Paola Fioretto, Maurizio Rossini, Alessandro Vignali, Fabrizio Fabris, Francesco Bertoldo

**Affiliations:** 1 Clinica Medica 1, Department of Medicine, University of Padova, 35128 Padova, Italy; stefania.sella@unipd.it (S.S.); dante.lucia11@gmail.com (M.F.); gparcidiacono91@gmail.com (G.A.); marcoonofrio.torres@studenti.unipd.it (M.O.T.); alberto.michielin89@gmail.com (A.M.); tancredi.prandini@gmail.com (T.P.); valeria.baffa@gmail.com (V.B.); andrea.aghi@gmail.com (A.A.); fabrizio.fabris@unipd.it (F.F.); 2 Unit of Clinica e Terapia Medica, Department of Medicine and Surgery, University of Parma, 43126 Parma, Italy; giovanni.passeri@unipr.it (G.P.); alessandro.vignali@gmail.com (A.V.); 3 National Research Council (CNR), Institute of Clinical Physiology (IFC), Clinical Epidemiology of Renal Diseases and Hypertension, Ospedali Riuniti, 89124 Reggio Calabria, Italy; gtripepi@ifc.cnr.it; 4 National Research Council (CNR), Institute of Clinical Physiology (IFC), 56124 Pisa, Italy; 5 CE Medical Writing, 56021 Pisa, Italy; colingegan@gmail.com; 6 Internal Medicine, Department of Medicine, University Hospital AOUI, 37134 Verona, Italy; martina.brigo@gmail.com (M.B.); francesco.bertoldo@univr.it (F.B.); 7 Laboratory Medicine Unit, Department of Medicine, University of Padova, 35128 Padova, Italy; marti.zaninotto@libero.it (M.Z.); mario.plebani@unipd.it (M.P.); 8 Clinica Medica 3, Department of Medicine, University of Padova, 35128 Padova, Italy; roberto.vettor@unipd.it (R.V.); paola.fioretto@unipd.it (P.F.); 9 Rheumatology Unit, Department of Medicine, University of Verona, 37134 Verona, Italy; maurizio.rossini@univr.it

**Keywords:** COVID-19, cholecalciferol, vitamin D, comorbid disease, mortality, intensive care, propensity score

## Abstract

Little information is available on the beneficial effects of cholecalciferol treatment in comorbid patients hospitalized for COVID-19. The aim of this study was to retrospectively examine the clinical outcome of patients receiving in-hospital high-dose bolus cholecalciferol. Patients with a positive diagnosis of SARS-CoV-2 and overt COVID-19, hospitalized from 15 March to 20 April 2020, were considered. Based on clinical characteristics, they were supplemented (or not) with 400,000 IU bolus oral cholecalciferol (200,000 IU administered in two consecutive days) and the composite outcome (transfer to intensive care unit; ICU and/or death) was recorded. Ninety-one patients (aged 74 ± 13 years) with COVID-19 were included in this retrospective study. Fifty (54.9%) patients presented with two or more comorbid diseases. Based on the decision of the referring physician, 36 (39.6%) patients were treated with vitamin D. Receiver operating characteristic curve analysis revealed a significant predictive power of the four variables: (a) low (<50 nmol/L) 25(OH) vitamin D levels, (b) current cigarette smoking, (c) elevated D-dimer levels (d) and the presence of comorbid diseases, to explain the decision to administer vitamin D (area under the curve = 0.77, 95% CI: 0.67–0.87, *p* < 0.0001). Over the follow-up period (14 ± 10 days), 27 (29.7%) patients were transferred to the ICU and 22 (24.2%) died (16 prior to ICU and six in ICU). Overall, 43 (47.3%) patients experienced the combined endpoint of transfer to ICU and/or death. Logistic regression analyses revealed that the comorbidity burden significantly modified the effect of vitamin D treatment on the study outcome, both in crude (*p* = 0.033) and propensity score-adjusted analyses (*p* = 0.039), so the positive effect of high-dose cholecalciferol on the combined endpoint was significantly amplified with increasing comorbidity burden. This hypothesis-generating study warrants the formal evaluation (i.e., clinical trial) of the potential benefit that cholecalciferol can offer in these comorbid COVID-19 patients.

## 1. Introduction

The first cases of serious coronavirus disease-19 (COVID-19) secondary to acute respiratory syndrome-coronavirus-2 (SARS-CoV-2) infection were reported in Wuhan, Hubei Province of China in January 2020 [1] where it subsequently spread worldwide [2,3] and was officially recognized as a pandemic by the WHO on 11th March 2020 [4].

Patients with COVID-19 typically show clinical signs of severe respiratory illness manifestations including fever, non-productive cough, dyspnea, myalgia, fatigue, abnormal leukocyte counts and radiographic evidence of interstitial pneumonia, symptoms similar to the well-known SARS-CoV and MERS-CoV infections [5].

SARS-CoV-2 infection can remain asymptomatic or cause modest symptoms. Severely sick patients require hospital admission and about 20% of them will develop acute respiratory distress syndrome (ARDS) and require intensive care unit (ICU) treatment [6] with increased risk of mortality [7,8], particularly in individuals with underlying comorbid diseases aged ≥60 years [9].

As of 16th December 2020, 1.64 million deaths have been reported worldwide with 68 million individuals testing positive for COVID-19 virus, 107,263 are in serious/critical condition and approximately 12,000 deaths are recorded daily worldwide. 

Italy was one of the first countries affected outside Asia and to date is one of the most affected countries in Europe alongside the United Kingdom, France and Spain where between 48,000 and 65,000 deaths have been reported for each of these countries as of 16th December 2020 [10]. Following a first wave of infection at the end of February 2020 and decreasing by the end of May 2020, many countries around the world are now experiencing a second one, accompanied with a high number of new cases daily and increasing number of deaths.

At the time when this paper was written, several COVID-19 vaccines were under development and interim results looked promising [11], however, their widespread use and related benefit was not expected to be realized in the near future. 

In the absence of effective therapies or vaccines, the medical and scientific community have extensively explored a range of currently available therapeutic agents, mainly focused on targeting viral replication as well as managing severe respiratory symptoms associated with COVID-19 [12].

In this regard, the activation of the vitamin D receptor (VDR) signaling pathway may generate beneficial effects in ARDS [13] by decreasing the cytokine/chemokine storm [14], thus having an important immunomodulatory and anti-inflammatory role [15].

To date, the protective properties of vitamin D supplementation have indeed been supported by numerous observational studies, and by meta-analysis of clinical trials for the prevention of viral acute respiratory infection [16]. In fact, insufficient vitamin D status has been suggested as a potential risk factor for non-communicable [17] and acute respiratory tract diseases [18,19], including viral infection [20].

Low vitamin D status (i.e., <50 nmol/L or 20 ng/mL [21] and measured as the plasma level of the transport form of vitamin D, 25(OH)D), is widespread worldwide and mainly seen in regions of northern latitudes, but also in southern countries [22]. For example, in Italy, the prevalence of low vitamin D is high in elderly women (aged 60–80 years) with values of 25(OH) vitamin D <5 ng/mL seen in 27% and <12 ng/mL in 76% [23]. This has prompted the question as to whether insufficient vitamin D could influence the course of COVID-19 [24,25,26]. Supporting other evidence [27], Ilie et al. recently analyzed data from 20 different European countries and observed a negative correlation (*r* = −0.44, *p* = 0.05) between levels of mean vitamin D (average 56.79 ± 10.61 nmol/L) and the number of cases of COVID-19/1 million population in each country [28]. The SENECA study group observed that mean vitamin D levels were severely low in the aging population, mainly in Spain (26 nmol/L), Italy (28 nmol/L) and Switzerland (23 nmol/L), this group recognized as the most vulnerable in relation to COVID-19 [29].

Although the benefit of supplementary vitamin D in patients with COVID-19 remains speculative and partially supported by limited data from observational studies [30] and three clinical trials [31,32,33], the real-life impact of vitamin D supplementation in patients infected with COVID-19 on outcome in Italy are still not available, particularly in elderly fragile patients with underlying comorbid diseases, who are at greatest risk for more severe clinical outcomes [34]. Furthermore, several studies evaluating the effect of vitamin D on COVID-19 patients have been limited by the low dose administered [30,31] or because patient characteristics may have precluded the possibility to observe a measurable effect of treatment [31]. The use of high-dose (~500,000 IU) cholecalciferol has already been shown to reduce hospital mortality in critically ill patients who were severely deficient in vitamin D [35], however, few studies have examined the effect of high-dose cholecalciferol in COVID-19 patients. The principal aim of this retrospective analysis was to evaluate the combined outcome of transfer to ICU and/or death in hospitalized comorbid fragile COVID-19 patients receiving 400,000 IU cholecalciferol (200,000 IU administered in two consecutive days) and also to determine if the effect of vitamin D may be modified by the comorbidity burden of these patients. In this regard, patients were also divided into four subgroups according to their comorbidity burden (no comorbidities, one comorbidity, two comorbidities and three or more comorbidities).

## 2. Materials and Methods

### 2.1. Study Population

Patients consecutively admitted from the emergency department in the internal medicine ward specifically dedicated to the management of COVID-19 patients in the University Hospital of Padova, Italy, from 15th March to 20th April 2020 were included in this retrospective analysis. All participants included in this study had: (1) a confirmed diagnosis of SARS-CoV-2 infection according to the WHO, by a positive result of real-time reverse transcriptase PCR (RT-PCR) assay on a nasopharyngeal swab [36]; (2) aged 40 or more years; (3) the presence on admission of fever (body temperature of ≥37.5 °C), interstitial lung pattern or consolidation on chest X-ray and dyspnea; (4) testing of serum 25(OH) vitamin D levels within 24 h of admission; (5) fully available data on the specific treatment applied, including in-hospital cholecalciferol administration. Exclusion criteria were: vitamin D supplementation within the past 2 months (from clinical history), presence of stage 5D chronic kidney disease (CKD), acute or chronic liver failure and concomitant use of enteral or parenteral nutrition. Based on these clinical characteristics, 91 patients (50 males and 41 females and aged 40–98 years) were considered in this study.

### 2.2. Data Collection

Computerized medical records were reviewed and demographic, clinical, radiological and laboratory data were collected at admission. These included the following: age, sex, body mass index (BMI), comorbidities (cardiovascular diseases (CVDs), diabetes mellitus, chronic obstructive pulmonary disease (COPD), endocrine diseases, CKD, active cancer, hematological diseases), current smoking status, vital signs, including pulse oxygen saturation (SpO_2_%), and, among a range of laboratory tests, serum 25(OH) vitamin D, serum calcium and phosphate, serum creatinine, serum C-reactive protein (CRP), serum procalcitonin (PCT), lactate dehydrogenase (LDH) and D-dimer. Partial pressure of oxygen (PaO_2_), fraction of inspired oxygen (FiO_2_) and their ratio (PaO_2_/FiO_2_) on arterial blood gas (ABG) were also collected.

### 2.3. Laboratory Analysis and X-ray

A standard X-ray image of the chest was obtained in all patients at admission. Fasting venous blood samples were collected from all patients for routine biochemistry analysis at the Department of Laboratory Medicine of the University of Padova, using accredited laboratory methods.

Serum 25(OH)D was measured using the automated immunochemiluminescent method, with a LIAISON^®^ 25 OH Vitamin D TOTAL Assay 310600 (DiaSorin Inc., Stillwater, MN, USA). Sensitivity was <10 nmol/L, and the intra-assay coefficients of variation (CV) were between 2.9% and 5.5%, while inter-assay CV was 6.3–12.9%. Both serum calcium and phosphate were measured using an automated colorimetric method on a Cobas 8000 (Roche Diagnostics, Mannheim, Germany), whereas serum creatinine was measured by an enzymatic method with calibration traceable to a reference procedure (isotope dilution-mass spectrometry) and automatized on a Cobas 8000. Serum CRP was measured by immunonephelometric assay automatized on a Dimension Vista (Siemens Healthcare GmbH, Eschborn, Germany) and serum PCT was measured by an immunochemiluminescent method (Liaison Brahms PCT II gen, Diasorin SpA, Saluggia, Italy). LDH was measured by spectrophotometric assay traceable to a method recommended by the International Federation of Clinical Chemistry automatized on a Cobas 8000. Serum D-dimer levels were measured by a turbidimetric assay on a Sysmex CS 5100 (Siemens Healthcare Diagnostics, Erlangen, Germany) and ABG analysis was performed by arterial point-of-care testing (POCT) ABG using a RAPIDPoint^®^ 405 (Siemens Healthcare Diagnostics, Erlangen, Germany).

### 2.4. Treatment

All patients received the best available treatment at that time according to clinical judgment (a variable combination of hydroxychloroquine, glucocorticoids, tocilizumab, lopinavir/ritonavir, azithromycin and/or other antibiotics). Based on individual clinical judgement of the referring physician, patients were administered 400,000 IU vitamin D supplementation as bolus oral cholecalciferol (two vials of 100,000 IU; DiBase, Abiogen Pharma, Pisa, Italy) daily for two consecutive days (the second and third day of the in-hospital stay). Following this treatment decision, patients were divided into two groups depending on whether they received cholecalciferol (*n* = 36; 39.6%) or not (*n* = 55; 60.4%).

### 2.5. Outcome Measures

Patients were followed during their in-hospital stay for an average time of 14 ± 10 days (median 12 days; absolute range 2–51 days). The majority of patients (*n* = 85; 93%) had a follow-up of ≤1 month. The follow-up time of the remaining 6 patients was as follows: 35 days in 1 case, 40 days in 2 cases, 46 days in 1 case, 48 days in 1 case and 51 days in 1 case. For the scope of this analysis, a composite outcome, including the need for transfer to ICU (for invasive mechanical ventilation) and/or death from any cause, was considered. Data were collected on an electronic case report form using Research Electronic Data Capture Software (REDCap) (Department of Medicine of the University of Padova).

All clinical investigations were conducted according to the principles expressed in the Declaration of Helsinki. Verbal consent from patients for the management of personal data was obtained. Permission for this retrospective data analysis was obtained from the Local Ethics Committee on 23/07/2020, CESC code: 6n/AO/20, protocol number: 0048697).

### 2.6. Statistical Analysis

Normally distributed continuous variables were summarized as mean and standard deviation, whereas non-normally distributed continuous variables were summarized as median and interquartile range. Binary and ordinal variables were expressed as absolute numbers and percentages. Between-group comparisons were performed by P for trend.

Receiver operating characteristic (ROC) curve analysis (expressed as area under the curve (AUC), odds ratio (OR) and respective 95% confidence intervals (CIs) was used to assess the predictive power of those variables able to better explain the decision of the physician to administer vitamin D. These variables were then also considered in a propensity score (propensity score 1). Levels of 25(OH) vitamin D and D-dimer were introduced as continuous variables into the model to derive the propensity score.

The effectiveness of vitamin D treatment on the incidence of the composite outcome was investigated by univariate and multiple logistic regression analyses. In multiple logistic regression models, with vitamin D as the key determinant and the incidence of the composite outcome as the dependent variable, the comorbidity burden was tested either as a potential confounder or as an effect modifier. The effect modification by the comorbidity burden on the relationship between vitamin D treatment and the composite outcome was analyzed by simultaneously introducing into the same multiple logistic regression model the treatment with vitamin D (0 = no; 1 = yes), the comorbidity burden (0, 1, 2 and ≥3) and their interaction term (i.e., vitamin D treatment × comorbidity burden). The ORs (and respective 95% CIs) of vitamin D treatment for the composite outcome across a different number of comorbidities (0, 1, 2 and ≥3) were calculated by the linear combination method and compared by the *p*-value for effect modification. In logistic regression analyses, data were expressed as OR, 95% CIs and *p*-value. The propensity score excluding the comorbidity burden (propensity score 2) was calculated only on the basis of factors (i.e., confounders) which significantly differed between vitamin D-treated and untreated patients. By this strategy, we constructed models of adequate statistical power (at least 10 patients with the event of interest for each covariate included in the model). Kaplan–Meier analysis and the Cox regression method were also applied as sensitivity analyses. In Cox regression models, data were expressed as hazard ratio (HR), 95% CIs and *p*-value. All calculations were performed by commercially available statistical software (STATA 13 StataCorp, Lakeway Drive, College Station, TX, USA).

## 3. Results

### 3.1. Patient Characteristics

**Figure 1:**
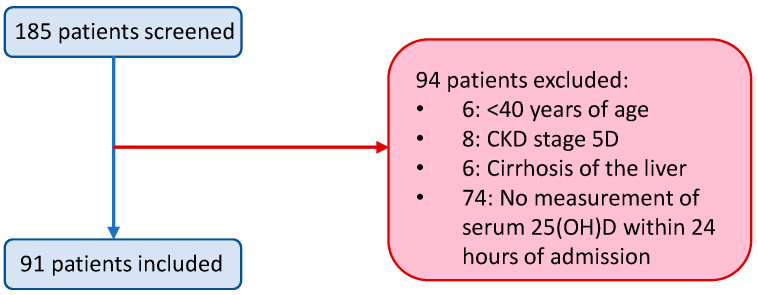
Patients initially screened, those excluded and included in the study. CKD = chronic kidney disease.

A total of 91 patients were included in this retrospective analysis. One hundred eighty-five patients were initially screened and 94 were excluded (Figure 1).

Thirty-six (39.6%) patients were administered 400,000 IU bolus cholecalciferol (200,000 IU administered in two consecutive days) and the remaining 55 (60.4%) patients received the best available treatment but were not administered native vitamin D. All patients were followed for an average of 14 ± 10 days.

Baseline characteristics for the total population as well as patients stratified by vitamin D treatment are summarized in Table 1. The majority of patients were elderly (mean age of 74 ± 13 years, ranging from 40–98 years) and 55% were male. Fifty (54.9%) patients presented with two or more comorbid diseases, the most frequent being cardiovascular disease (79%) and diabetes (33%). A higher proportion of patients subsequently treated with vitamin D were current smokers (36 vs. 15%, *p* = 0.018) and had a higher burden of comorbidities (*p* = 0.013); 70% having two or more comorbidities vs. 45% in patients without vitamin D; *p* = 0.042. Furthermore, 25(OH) vitamin D levels were significantly lower in patients treated with vitamin D (24 nmol/L vs. 36 nmol/L, *p* = 0.015) and D-dimer levels were also three-fold higher in patients treated with vitamin D (936 vs. 317 µg/L, *p* = 0.001). Among other best available treatments administered to these critically ill patients, the anti-malarial drug hydroxychloroquine was administered to a greater extent in vitamin D-treated patients (92% vs. 64%, *p* = 0.003) in addition to a lopinavir/ritonavir antiviral combination (58% vs. 16%, *p* < 0.001), with a lower proportion receiving other antibiotics (67% vs. 86%, *p* = 0.035).

### 3.2. Drivers of Vitamin D Treatment

**Figure 2:**
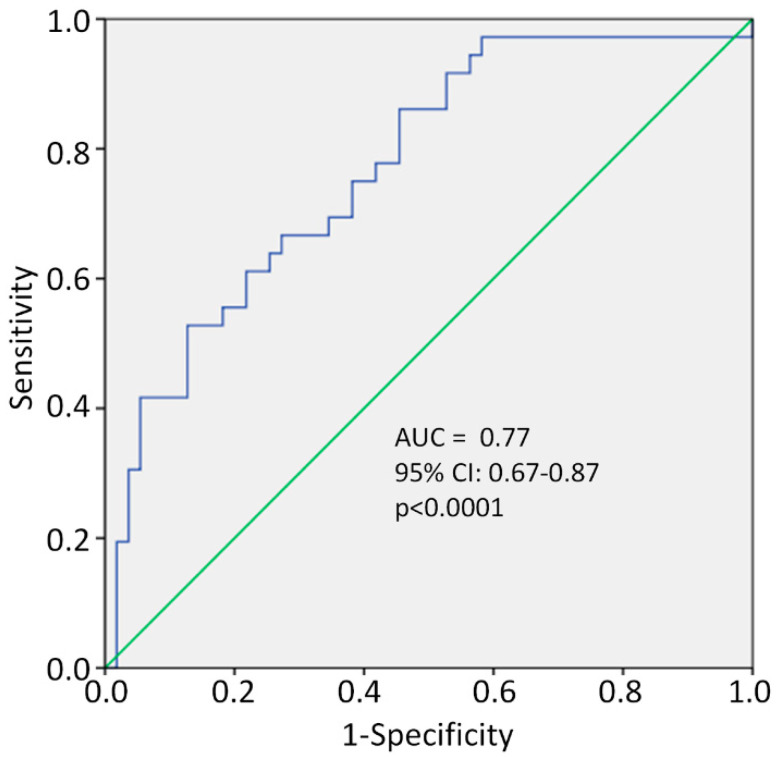
Receiver operating characteristic (ROC) curve of propensity score for vitamin D treatment, including smoking, serum D-dimer and 25(OH)D levels and comorbidity burden. AUC = area under the curve, CI = confidence interval.

The decision to treat with vitamin D was clinical and at the discretion of the individual treating physician who visited each patient. However, when retrospectively looking at the data, the propensity to treat patients was essentially based on the same characteristics found to be significantly different between the two groups (Table 1). These included: (1) patients presenting with low serum 25(OH) vitamin D levels (i.e., <50 nmol/L [21]), (2) being a current cigarette smoker, (3) elevated serum D-dimer levels and (4) the presence of comorbid diseases. ROC analysis revealed a significant predictive power of these four variables (jointly considered in propensity score 1); these four variables accounted for 77% of the propensity (decision) of the physician to administer vitamin D (AUC = 0.77, 95% CI: 0.67–0.87, *p* < 0.0001; Figure 2).

### 3.3. Association between Vitamin D Treatment and Clinical Outcome

We next examined the association between vitamin D treatment and clinical outcome. For this analysis, patients were divided into four groups according to the number of underlying comorbidities (no comorbidities, one comorbidity, two comorbidities and three or more comorbidities) (Table 2). The most frequent comorbidities were CVD (79%), followed by diabetes (33%). A total of 26 (28.6%) patients were burdened with three or more comorbid diseases and in this group of patients, 96% had CVD, 65% had diabetes and 39% had CKD, with significantly higher D-dimer levels (1.165 µg/L) compared to patients with one or two comorbidities (544 µg/L) or no comorbidities (234 µg/L). No differences were observed among a range of other clinical and laboratory variables collected (Table 2). 

The primary endpoint in this study was a composite outcome of death from any cause and/or need for transfer to an ICU over an average of a 14 ± 10 day follow-up period after hospital admission. The single need for transfer to ICU was invasive mechanical ventilation. Twenty-seven (29.7%) patients were transferred to the ICU and 22 (24.2%) patients died, in six cases after being transferred to the ICU (in 16 cases prior to transfer to ICU). Overall, 43 (47.3%) patients experienced the combined endpoint of transfer to ICU or death. In a crude analysis, initially including comorbidity burden as a potential confounder, vitamin D treatment was observed to be associated with a 43% and 55% reduction, respectively, in the OR of the combined endpoint, but these effects did not attain statistical significance (Table 3).

However, our analysis revealed that when the comorbidity burden was considered as an effect modifier (rather than as a potential confounder) a significant effect modification by this variable was found for the vitamin D outcome linked to both the crude (*p* = 0.033) and propensity score 2-adjusted analysis (*p* = 0.039) (Table 4). Of note, this effect modification remained significant (*p* = 0.018) after also forcing age and treatment with tocilizumab into the logistic model (e.g., two variables which were not specifically included in the propensity score, but were different across patient stratification). This analysis showed that the OR of vitamin D treatment for the incidence of the combined outcome was reduced in parallel with the increase in the comorbidity burden (Table 4).

A sensitivity analysis using the Kaplan–Meier method showed that the in-hospital mortality rate did not significantly differ between vitamin D-treated and untreated patients (see Supplementary Materials Figure S1). However, when mortality was jointly considered with ICU transfer within a composite endpoint, both Kaplan–Meier survival curves (see Supplementary Materials Figure S2, upper panel) and Cox regression analyses (see Supplementary Materials Figure S2, bottom panel) confirmed a significant effect modification by the comorbidity burden on the effectiveness of vitamin D on this combined endpoint.

## 4. Discussion

In the present study, we observed that the comorbidity burden significantly modified the effect of vitamin D treatment on the incidence of the composite outcome of death from any cause and/or need for transfer to the ICU in COVID-19 patients, the OR of vitamin D treatment for the incidence of this composite endpoint being reduced in parallel with the increase in the number of comorbidities (see Table 4). The action of the comorbidity burden as an effect modifier is further corroborated by the observation that simple data adjustment for this risk factor (i.e., by considering background comorbidities as a potential confounder) (see Table 3) did not affect the strength of the relationship between vitamin D treatment and the study outcome.

In the midst of a second wave of the COVID-19 pandemic with high death tolls registered worldwide [10], the unavailability of vaccines (at the time when this paper was written) and ineffective “best treatment options” have driven clinicians to evaluate a wide range of currently available therapies that may offer hope in the management ARDS associated with COVID-19 [12].

Thanks to its pleiotropic actions, vitamin D has recently emerged as one such hopeful candidate through its established ability to decrease the cytokine/chemokine storm [14] via immunomodulatory [37] and anti-inflammatory effects [15] that could well result in a clinically important benefit in COVID-19 patients with ARDS [13,38].

This present analysis follows in the footsteps of tentative evidence derived from a recent prospective study [30] and three clinical trials [31,32,33], two of which have shown benefit (one did not observe any effect) in patients hospitalized with COVID-19 following vitamin D supplementation.

The rationale for these studies, and ours, was primarily based on evidence derived from studies showing that patients with hypovitaminosis D had an increased risk of being infected with SARS-CoV-2 [39] and that cases with COVID-19 had lower serum 25(OH)D levels compared to controls without COVID-19 [40]. Other studies have shown that patients with lower levels of vitamin D had worse outcomes [41,42,43], including a higher risk of mortality [28], which was also confirmed in a meta-analysis [44].

In this retrospective study involving 91 patients affected by COVID-19, we treated 36 (39.6%) patients considered to be at the greatest risk of a serious outcome (based on serum 25(OH)D levels, D-dimer levels, smoking status and comorbid burden) with high doses (400,000 IU) of oral cholecalciferol. During a follow-up period of approximately two weeks, 27 (29.7%) patients were transferred to the ICU and 22 (24.2%) died.

Patients hospitalized in this study were extremely fragile, in that they presented with advanced age (mean age of 74 years) and 79% had CVD and 23% were current cigarette smokers. Moreover, the burden of these and other comorbid diseases fits with the profile typically observed in these patients [34].

Furthermore, ROC curve analysis revealed a significant predictive power of the four variables: (a) low 25(OH) vitamin D levels, (b) current smoker, (c) elevated D-dimer levels and (d) comorbid diseases to explain the decision to administer vitamin D.

While low 25(OH) vitamin D [28,39,40,41,42,44], high D-dimer levels [45] and the presence of comorbid diseases [34] are associated with poorer outcome in patients hospitalized with COVID-19, the precise role of smoking status has remained under debate [46,47], even though an association between smoking status and vitamin D [48] is recognized.

D-dimer is a fibrin degradation product which is increased in thrombotic events, indicating fibrinolysis [49]. High values of D-dimer are associated with the activation of the coagulation cascade secondary to systemic inflammatory response syndrome in COVID-19 patients [50], fitting with observations from our study and others [51] that elevated D-dimer levels are associated with worse outcomes in COVID-19 patients.

The association between vitamin D and CKD is well established [52,53]. However, sensitivity analysis did not identify an effect modification by CKD on the vitamin D–study outcome link. The severity of CKD in patients included in this analysis (no patients had stage 5 CKD) was unlikely to have influenced the normal rate of conversion of 25(OH)D to 1,25(OHD).

It is important to highlight that the beneficial effect of cholecalciferol on outcome (transfer to ICU or death) was gradually more pronounced with increasing comorbidity burden and this was true for both crude and propensity-adjusted analyses (Table 4).

Little evidence from observational studies and trials to date have evaluated the effect of high-dose cholecalciferol in hospitalized COVID-19 patients.

In the quasi-experimental prospective study by Annweiler and colleagues, patients hospitalized for proven COVID-19 were stratified into three groups: Group 1, in which vitamin D was regularly supplemented during the 12 months prior to diagnosis; Group 2 in which vitamin D was given soon after hospital admission and Group 3, in which patients were not treated with vitamin D. Those who were supplemented with vitamin D after a diagnosis of COVID-19 did not show any significant benefit compared to untreated controls [30]. Instead, patients who regularly received bolus vitamin D3 supplementation in the preceding 12 months experienced a significantly lower mortality rate over 14 days (HR: 0.07, *p* = 0.017) compared to the control group (without vitamin D). Patients in the second group (receiving vitamin D after COVID-19 diagnosis) were older than in our study (85 vs. 74 years) with higher male predominance (68.7% vs. 53% in our study), and higher CRP levels (69 vs. 55 mg/L), characteristics which would not explain the lack of benefit compared to the control group. However, the dose of oral vitamin D3 was 80,000 IU, approximately five times lower than that administered to patients in the present study, potentially a decisive explanatory factor for the differences observed. In a randomized controlled trial performed in India on 40 middle-aged patients positive for COVID-19, 16 patients were randomized to receive bolus cholecalciferol (80,000 IU for 7 days) and 24 patients did not receive vitamin D (control group) [32]. Although mortality was not assessed in that trial, 10/16 (62.5%) patients receiving a high dose of cholecalciferol (admittedly spread over 7 days), similar to that used in the present study (560,000 IU vs. 400,000 IU), achieved SARS-CoV-2 negativity compared to five out of 24 (20.8%) participants (*p* = 0.018) in the control arm. Furthermore, a 42.4 ng/mL increase in serum 25(OH)D was seen after 14 days (compared to a 5.1 ng/mL increase in the placebo group). This trial included patients aged around 50 years and as such are poorly representative of patients hospitalized with COVID-19 in Europe. Furthermore, no effort to adjust for confounding variables was considered in their analysis. In another randomized controlled trial involving 240 patients hospitalized with severe COVID-19, a single dose of 200,000 IU of vitamin D3 supplementation was found to be effective in increasing 25(OH) vitamin D levels, but it did not significantly decrease length of hospital stay or other clinical outcomes compared to the placebo group. Patients were aged 56.8 years and 57.8% were obese (and 91.7% obese or overweight), characteristics again that are poorly representative of patients in Europe, but have been observed elsewhere [54]. The reduced age, high frequency of obesity and only mildly reduced basal 25(OH)D levels (21 ng/mL) may have precluded the possibility to observe a clinically measurable effect of vitamin D.

Last, in a pilot randomized study undertaken in Spain, a high dose of calcifediol significantly reduced the need for ICU treatment in patients hospitalized for COVID-19 compared to an untreated control group [33]. Although this study was well designed, patient age (53 years) and the use of calcifediol make it difficult to compare results with our study.

Collectively, studies evaluating the effect of vitamin D on outcome in patients hospitalized for COVID-19 provide some cautious optimism for the benefit afforded by vitamin D, particularly at higher doses [32]. However, limitations in heterogenous patient populations and other design weaknesses preclude the generalizability of these studies.

The benefit and safety from administering a higher dose of cholecalciferol in critically ill patients is recognized [35] and support our findings and others [32] to also use this regime in patients with overt COVID-19.

Studies examining vitamin D status in Europe observe that vitamin D deficiency is more frequent in Southern than in Northern Europe, a rather unexpected finding considering that ultraviolet light exposure is higher in Southern than in Northern European countries [22]. This is particularly evident in the elderly population, such as women with osteoporosis, in countries like Italy and Greece [23,55,56]. Unfortunately, recent legislation in Italy has limited the use of vitamin D to specific high-risk groups [57]. Actually, in the first trimester of 2020 (just prior to the pandemic), Italy reported a 30% reduction in the use of vitamin D [58], in contrast to other countries such as the UK [59], where vitamin D is now administered free of charge to certain populations.

While findings from the present retrospective analysis should serve to promote discussion and be strictly regarded only as hypothesis generating, we would recommend that this high-risk fragile group of patients have their 25(OH)D status monitored and be administered high-dose cholecalciferol if they also present with high D-dimer and several comorbid diseases, along with current smoking status. Indeed, there would appear to be more to offer these critically ill patients by administering vitamin D than not.

## 5. Limitations

This study does have several limitations that should be mentioned. Observational studies involving databases that are retrospectively examined may be characterized by a lack of reliability of results and incompleteness of data, issues associated with this design that have been already established [60,61]. However, the added benefits of an observational design are being increasingly recognized, particularly with regard to evaluating the effect of supplements, such as vitamin D [62]. That said, the short follow-up time and close monitoring of these patients ensured that missing data were not relevant in this case.

This study included patients from a single center in Northern Italy (Padua) and as such our findings may not represent the entire Italian territory. At least, we cannot confirm that the response of patients hospitalized with COVID-19 to cholecalciferol would be the same across different Italian regions, where the characteristics of patients with COVID-19 can vary [2,63].

No formal sample size calculation was performed, since this retrospective analysis included patients who were consecutively hospitalized. The time needed to generate a larger sample size would have obviously conflicted with the urgent nature to communicate this hypothesis-generating study.

Serum calcium and phosphorus levels were not assessed during the follow-up period. However, evidence from previous studies has demonstrated that high doses of cholecalciferol were found to be safe, with no episodes of hypercalcemia seen [32,35,64].

In line with this, another limitation of this retrospective design was the fact that a post-dose assessment of serum 25(OH)D was not available. Despite this, extensive literature has already documented the normalization of serum 25(OH)D following high-dose cholecalciferol, including critically ill patients [35], as well as in patients hospitalized for COVID-19 [32].

We are aware that the Charlson comorbidity index (CCI) [65] is considered a good estimate of patients’ prognosis and the fact that it was not used in this study may be a potential limitation. However, it is mainly used to evaluate the 1-year mortality risk, not relevant for our study. Furthermore, in these critically ill patients, physicians were under extreme pressure to rapidly make a correct diagnosis and decide on how best to treat and manage the patient. Due to time restrictions in this real-life scenario, the completion of the CCI online form may not have been appropriate and we therefore used the number of comorbidities as a measure of prognosis.

Due to the limited number of patients included in this analysis (*n* = 91) as well as because of the relatively low number of patients with the combined endpoint (*n* = 43), we used a propensity score to adjust the interaction between comorbidity burden and vitamin D treatment as a determinant of transfer to ICU and/or death. The fact that all patients who participated in this study were naïve to recent vitamin D supplementation allowed us to avoid important confounding issues, such as confounding by indication, as well as residual confounding. Despite the limited number of patients included in this analysis, both crude and propensity score-adjusted analyses display similar values in terms of OR and *p*-value, providing some degree of internal consistency. More important, perhaps, is that the only other similar study performed to date examining the effect of high-dose vitamin D on outcome in patients hospitalized for COVID-19 [30] shows similar ORs for crude analysis.

## 6. Conclusions

Results from this retrospective analysis demonstrate that two consecutive doses of 200,000 IU cholecalciferol (total of 400,000 IU) can significantly improve the outcome in patients affected by COVID-19 that are also burdened with three or more comorbid diseases. These findings are hypothesis generating and as such should be treated with caution.

Further studies with a larger sample size and longer follow-up period to specifically examine the relative impact of individual comorbid conditions, such as CVD, COPD, diabetes, hematological diseases and particularly CKD, would have important clinical use. In our preliminary data analysis phase, we specifically tested the presence/absence of an effect modification due to each single comorbidity and we found no significant effect. Therefore, our study generates the hypothesis that when evaluating the effectiveness of vitamin D treatment in COVID-19 patients, clinicians should consider the burden of comorbidities rather than a single comorbidity.

## Figures and Tables

**Table 1:** Patient characteristics stratified by treatment with vitamin D.

Characteristic	Total Population (*n* = 91)	No Treatment with Vitamin D (*n* = 55)	Treatment with Vitamin D (*n* = 36)	*p*-Value
**General**				
Age (years)	74 ± 13	74 ± 13	73 ± 13	0.817
Male gender, n (%)	50 (55)	31 (56)	19 (53)	0.738
Current smokers, n (%)	21 (23)	8 (15)	13 (36)	**0.018**
BMI (kg/m^2^)	26.6 ± 4.2	27 ± 5	26 ± 4	0.099
**Comorbidity burden, n (%)**				
0	9 (10)	7 (13)	2 (6)	
1	32 (35)	23 (42)	9 (25)	**0.013**
2	24 (26)	14 (25)	10 (28)	
≥3	26 (29)	11 (20)	15 (42)	
**Major comorbidities, n (%)**				
Cardiovascular disease	72 (79)	43 (78)	29 (81)	0.786
COPD	17 (19)	7 (13)	10 (28)	0.073
CKD	13 (14)	2 (4)	11 (31)	**<0.001**
Active cancer	11 (12)	7 (13)	4 (11)	0.818
Diabetes mellitus	30 (33)	18 (33)	12 (33)	0.952
Hematological diseases	8 (9)	2 (4)	6 (17)	**0.033**
Endocrine diseases	14 (15)	7 (13)	7 (19)	0.388
**Respiratory function/laboratory**				
PaO_2_/FiO_2_ mmHg ratio	280 (188–336)	280 (173–328)	280 (192–357)	0.612
PaO_2_ (mmHg)	71 (65–81)	71 (65–81)	72 (63–83)	0.606
O_2_ saturation (%)	96 (95–98)	96 (95–98)	96 (93–97)	0.157
25(OH) vitamin D (nmol/L)	35 (16–60)	36 (19–77)	24 (12–42)	**0.015**
Creatinine (µmol/L)	88 (69–115)	88 (71–113)	88 (62–115)	0.812
Procalcitonin (µg/L)	0.25 (0.08–0.52)	0.21 (0.07–0.48)	0.41 (0.13–0.60)	0.490
D-dimer (µg/L)	610 (207–1264)	317 (172–966)	936 (533–1452)	**0.001**
LDH (U/L)	365 (240–484)	373 (264–503)	317 (218–394)	0.057
Calcium (mmol/L)	2.14 (2.07–2.21)	2.14 (2.07–2.24)	2.15 (2.07–2.19)	0.842
Phosphate (mmol/L)	0.95 (0.78–1.13)	0.93 (0.74–1.13)	0.97 (0.78–1.16)	0.167
CRP (mg/L)	64 (30–133)	69 (33–148)	55 (25–88)	0.091
**Current treatment, n (%)**				
Hydroxychloroquine	68 (75)	35 (64)	33 (92)	**0.003**
Azithromycin	70 (77)	39 (71)	31 (86)	0.094
Glucocorticoids	41 (45)	24 (44)	17 (47)	0.738
Tocilizumab	13 (14)	6 (11)	7 (19)	0.258
Lopinavir/ritonavir	30 (33)	9 (16)	21 (58)	**<0.001**
Other antibiotics	71 (78)	47 (86)	24 (67)	**0.035**

Data are expressed as mean ± standard deviation, median and interquartile range, or absolute number and percentage, as appropriate. BMI = body mass index, CKD = chronic kidney disease, COPD = chronic obstructive pulmonary disease, CRP = C-reactive protein, LDH = lactose dehydrogenase, PaO_2_/FiO_2_ = partial pressure of oxygen/fraction of inspired oxygen. The *p*-values in bold text denote statistically significant differences between specific variables according to treatment with vitamin D. Normal laboratory ranges can be found at the following link: https://emedicine.medscape.com/article/2172316-overview.

**Table 2:** Patient characteristics stratified by comorbidity burden.

Characteristics	Total Population (*n* = 91)	No Comorbidities (*n* = 9)	One Comorbidity (*n* = 32)	Two Comorbidities (*n* = 24)	≥3 Comorbidities (*n* = 26)	*p*-Value
**General**						
Age (years)	74 ± 13	63 ± 13	73 ± 14	77 ± 10	75 ± 12	**0.021**
Male gender, n (%)	50 (55)	7 (78)	17 (53)	12 (50)	14 (54)	0.416
Current smokers, n (%)	21 (23)	4 (44)	6 (19)	2 (8)	9 (35)	0.892
BMI (kg/m^2^)	26.6 ± 4.2	27.5 ± 2.7	26.6 ± 5.1	27.4 ± 4.0	25.6 ± 4.0	0.284
**Major comorbidities, n (%)**						
Cardiovascular disease	72 (79)	0 (0)	26 (81)	21 (88)	25 (96)	**<0.001**
COPD	17 (19)	0 (0)	0 (0)	8 (33)	9 (35)	**<0.001**
CKD	13 (14)	0 (0)	0 (0)	3 (13)	10 (39)	**<0.001**
Active cancer	11 (12)	0 (0)	1 (3)	2 (8)	8 (31)	**0.001**
Diabetes mellitus	30 (33)	0 (0)	4 (13)	9 (38)	17 (65)	**<0.001**
Hematological diseases	8 (9)	0 (0)	1 (3)	1 (4)	6 (23)	**0.008**
Endocrine diseases	14 (15)	0 (0)	0 (0)	4 (17)	10 (39)	**<0.001**
**Respiratory function/laboratory**						
PaO_2_/FiO_2_ mmHg ratio	280 (188–336)	323 (269–381)	295 (191–338)	277 (194–349)	214 (163–304)	0.067
PaO_2_ (mmHg)	71 (65–81)	79 (76–114)	73 (63–82)	70 (63–78)	66 (63–77)	0.062
O_2_ saturation (%)	96 (95–98)	98 (96–99)	96 (95–98)	96 (94–97)	97 (92–98)	0.612
25(OH) vitamin D (nmol/L)	35 (16–60)	49 (38–59)	24 (14–59)	32 (15–70)	36 (17–54)	0.417
Creatinine (µmol/L)	88 (69–115)	72 (65–95)	94 (77–116)	86 (73–116)	80 (64–127)	0.862
Procalcitonin (µg/L)	0.25 (0.08–0.52)	0.17 (0.10–0.96)	0.25 (0.07–0.64)	0.26 (0.08–0.41)	0.27 (0.10–1.20)	0.851
D-dimer (µg/L)	610 (207–1264)	234 (155–699)	544 (177–963)	544 (243–1029)	1165 (513–2314)	**0.001**
LDH (U/L)	365 (240–484)	239 (217–382)	372 (269–483)	316 (250–483)	391 (227–559)	0.134
Calcium (mmol/L)	2.14 (2.07–2.21)	2.14 (1.97–2.19)	2.15 (2.07–2.24)	2.14 (2.08–2.19)	2.12 (2.00–2.22)	0.166
Phosphate (mmol/L)	0.95 (0.78–1.13)	0.79 (0.58–0.97)	0.95 (0.78–1.13)	0.97 (0.92–1.24)	0.90 (0.65–1.16)	0.307
CRP (mg/L)	64 (30–133)	54 (33–125)	86 (40–193)	62 (23–145)	56 (23–87)	0.134
**Current treatment, n (%)**						
Hydroxychloroquine	68 (75)	7 (78)	22 (69)	19 (79)	20 (77)	0.636
Azithromycin	70 (77)	6 (67)	26 (81)	20 (83)	18 (69)	0.698
Glucocorticoids	41(45)	2 (22)	13 (41)	13 (54)	13 (50)	0.146
Tocilizumab	13 (14)	0 (0)	2 (6)	4 (17)	7 (27)	**0.011**
Lopinavir/ritonavir	30 (33)	3 (33)	7 (22)	7 (29)	13 (50)	0.074
Other antibiotics	71 (78)	7 (78)	25 (78)	19 (79)	20 (77)	0.944
Cholecalciferol	36 (40)	2 (22)	9 (28)	10 (42)	15 (58)	**0.013**

Data are expressed as mean ± standard deviation, median and interquartile range, or absolute number and percentage, as appropriate. BMI = body mass index, CKD = chronic kidney disease, COPD = chronic obstructive pulmonary disease, CRP = C-reactive protein, LDH = lactose dehydrogenase, PaO_2_/FiO_2_ = partial pressure of oxygen/fraction inspired oxygen. The *p*-values in bold text denote statistically significant differences among specific variables between groups of patients according to comorbidity burden. Normal laboratory ranges can be found at the following link: https://emedicine.medscape.com/article/2172316-overview.

**Table 3:** Crude and adjusted logistic regression analyses to examine the association between vitamin D and study outcome.

Variables		Crude Analyses (OR, 95% CI and *p*-Value)	Model 1 (OR, 95% CI and *p*-Value)
Vitamin D treatment	(0 = no; 1 = yes)	0.57 (0.24–1.34), *p* = 0.20	0.45 (0.20–1.22), *p* = 0.13
Comorbidity burden	(1-unit increase)	1.17 (0.76–1.78), *p* = 0.48	1.29 (0.82–2.01), *p* = 0.27

In model 1, the comorbidity burden is considered as a potential confounder. OR = odds ratio, CI = confidence interval.

**Table 4:** Logistic regression analysis to examine the effect modification by the comorbidity burden on the effectiveness of vitamin D on the study outcome.

Comorbidity Burden	Crude Effect Modification Analysis * (OR, 95% CI and *p*-Value)	Adjusted Effect Modification Analysis ** (OR, 95% CI and *p*-Value)
0	3.26 (0.48–22.2)	3.59 (0.52–24.8)
1	1.16 (0.36–3.76)	1.32 (0.39–4.43)
2	0.41 (0.16–1.05)	0.49 (0.18–1.32)
≥3	0.15 (0.034–0.64)	0.18 (0.04–0.83)
P for effect modification by comorbidity burden	**0.033**	**0.039**

* Adjusted for the main effect of vitamin D treatment and comorbidity burden. ** Adjusted for the main effect of vitamin D treatment, comorbidity burden and propensity score 2. OR = odds ratio, CI = confidence interval. *p*-value < 0.05 in bold.

## Data Availability

The data presented in this study are available on request from the corresponding author.

## Supplementary Materials

The following are available online at https://www.mdpi.com/2072-6643/13/1/219/s1, Figure S1: Kaplan–Meier survival curve of in-hospital mortality in vitamin D-treated and untreated patients, Figure S2: Kaplan–Meier survival curves of the effect modification by comorbidity burden on the effectiveness of vitamin D treatment for the combined endpoint (death/ICU transfer).
