# Supplementary material for: Effectiveness of In-Hospital Cholecalciferol Use on Clinical Outcomes in Comorbid COVID-19 Patients: A Hypothesis-Generating Study
Source: Nutrients. 2021 Jan 14;13(1):219. doi: 10.3390/nu13010219 (PMC7828675; doi:10.3390/nu13010219)
Supplement: Supplementary file 1 [file nutrients-13-00219-s001.zip › nutrients-1059418-supplementary.pptx]

## Slide 1
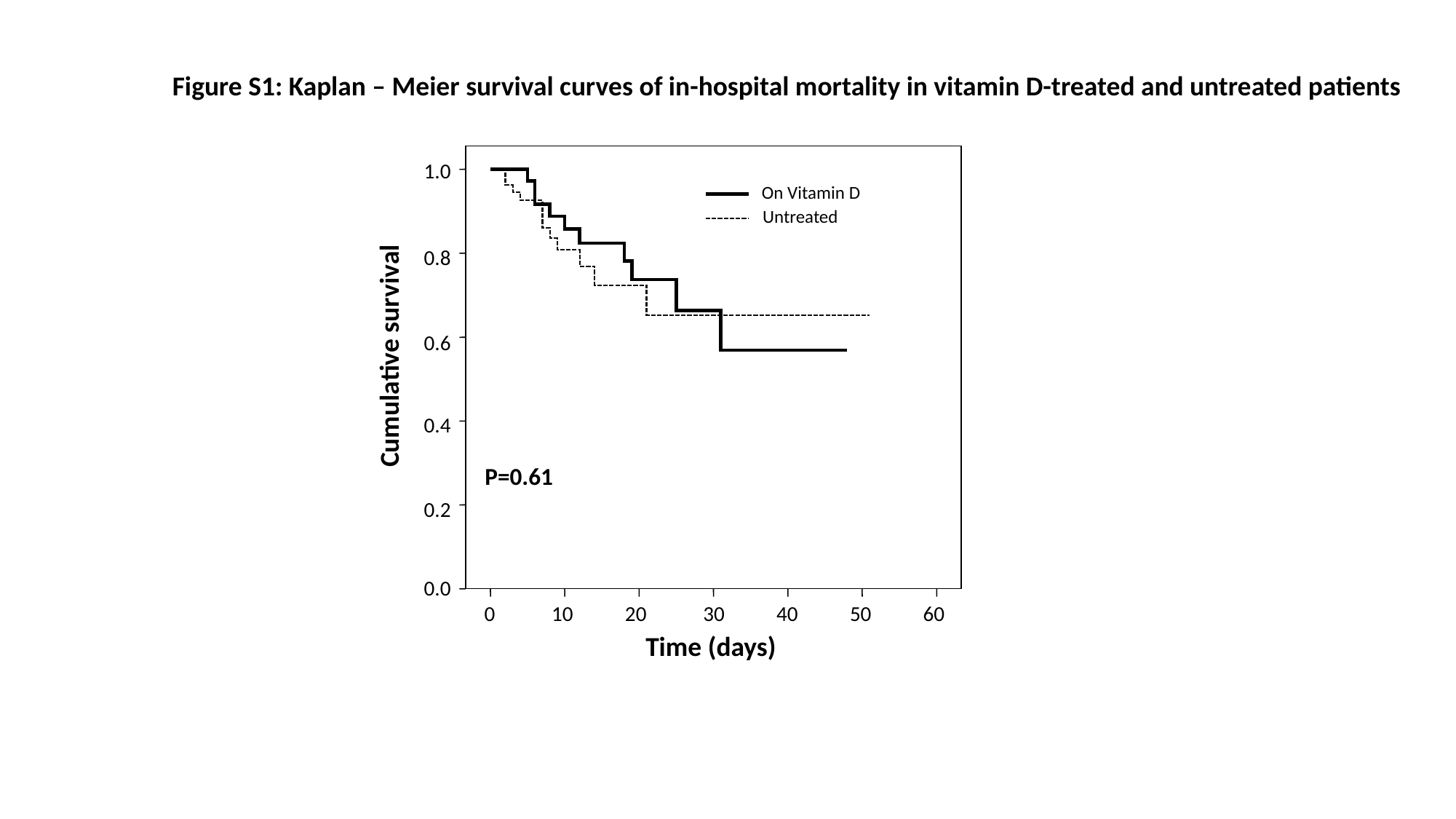

Figure S1: Kaplan – Meier survival curves of in-hospital mortality in vitamin D-treated and untreated patients
1.0
On Vitamin D
Untreated
0.8
0.6
Cumulative survival
0.4
P=0.61
0.2
0.0
0 10 20 30 40 50 60
Time (days)

## Slide 2
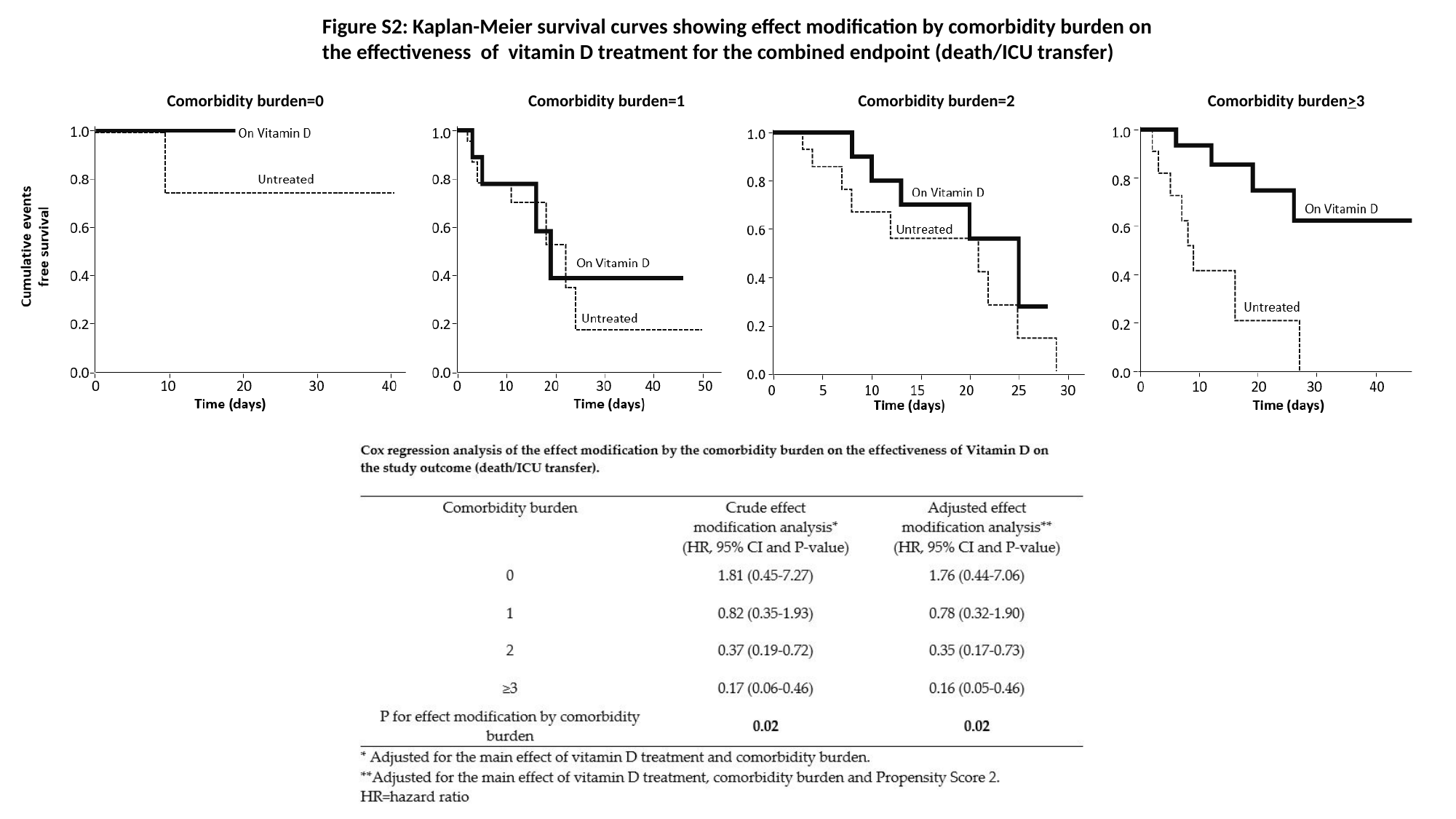

Figure S2: Kaplan-Meier survival curves showing effect modification by comorbidity burden on
the effectiveness of vitamin D treatment for the combined endpoint (death/ICU transfer)
Comorbidity burden=0
Comorbidity burden=1
Comorbidity burden=2
Comorbidity burden>3
